# Fungal Needle Communities and *Dothistroma septosporum* Occurrence in *Pinus sylvestris* Stands in Slovakia

**DOI:** 10.1002/ece3.74481

**Published:** 2026-10-16

**Authors:** Renata Artimová, Katarína Adamčíková, Juraj Medo, Marek Kobza, Emília Ondrušková, Zuzana Jánošíková

**Affiliations:** ^1^ Department of Plant Pathology and Mycology, Institute of Forest Ecology Slovak Academy of Sciences Nitra Slovakia; ^2^ Institute of Biotechnology, Faculty of Biotechnology and Food Science Slovak University of Agriculture in Nitra Nitra Slovakia

**Keywords:** *Dothistroma*, fungal communities, native stands, needle mycobiome, *Pinus sylvestris*

## Abstract

Dothistroma needle blight (DNB), caused by *Dothistroma septosporum*, is a globally important disease of pines, yet its ecological role within native host systems remains incompletely understood. We combined species‐specific quantitative PCR (qPCR) with ITS2 metabarcoding to investigate the occurrence of *D. septosporum* and associated needle mycobiomes of *Pinus sylvestris* in natural stands in Slovakia with a pooled non‐natural positive‐reference group included for comparison. *D. septosporum* was detected in the Vernár natural stand (11 of 16 samples), where visible DNB symptoms were also observed, and in all samples of the non‐natural positive‐reference group (15 of 15 samples). The pathogen was not detected in the other three sampled natural stands. Fungal community analyzes revealed significant differences in community composition among sample groups, while alpha diversity patterns showed comparatively minor variation. The most abundant genus across all sites was *Lophodermium*, whereas *Dothistroma* was highly abundant only in the non‐natural positive‐reference group. Co‐occurrence network analysis indicated that fungal communities were dominated by positive associations; however, patterns involving *Dothistroma* primarily reflected the positive‐reference group, where the pathogen was highly abundant. Our results suggest that *D. septosporum* is not a consistently detectable component of needle mycobiomes in native *P. sylvestris* stands in Slovakia and that its high abundance in the non‐natural positive‐reference group was associated with a distinct fungal community composition.

## Graphical abstract

Species‐specific quantitative PCR (qPCR) with ITS2 metabarcoding was combined to investigate the occurrence of *D. septosporum* and associated needle mycobiomes of *Pinus sylvestris* in natural and non‐natural stands in Slovakia. Fungal community analyzes revealed significant differences in community composition among stands, while alpha diversity patterns showed comparatively minor variation. Results suggest that *Dothistroma septosporum* is not a consistent component of needle mycobiomes in native *P. sylvestris* stands in Slovakia and that its proliferation is associated with altered, non‐natural systems rather than long‐term stable coexistence.

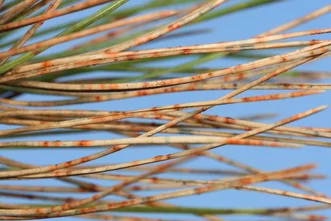

## 1 Introduction

The fungus *Dothistroma septosporum*, causing Dothistroma needle blight (DNB), is one of the most important pathogens of pine trees with a wide host range and global distribution in both the Northern and Southern Hemispheres (Drenkhan et al. 2016). The disease causes foliar necrosis, premature needle cast (Figure 1), reduced tree growth, and, in severe cases, tree mortality (Brown and Webber 2008) can result in substantial economic losses, primarily through reduced timber production and losses in forest plantations. In addition to its direct economic impacts on forestry, the disease can negatively affect the landscape and recreational value of forests, as well as the esthetic value of ornamental trees (Bulman 2007; Price 2010; Brown 2011). The worldwide distribution of this pathogen has initiated investigations regarding its historical origin and native range. The development of molecular markers, including microsatellite and mating type markers (Barnes et al. 2008; Groenewald et al. 2007), has enabled detailed analyzes of *D. septosporum* populations.

**FIGURE 1:**
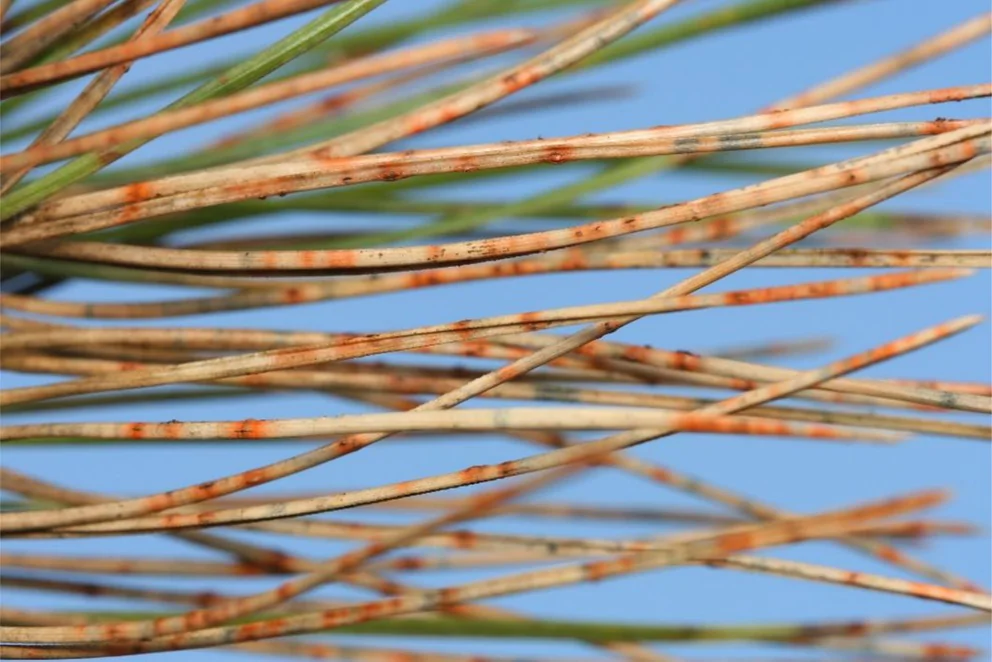
*Dothistroma septosporum* infection symptoms on *Pinus sylvestris* needles, showing characteristic reddish‐brown bands and dark fungal stromata visible within the affected areas.

Many studies have investigated the genetic diversity and reproductive structure of populations at national or regional scales (Dale et al. 2011; Drenkhan et al. 2013; Mullett et al. 2017; Boroń et al. 2019; Tomšovský et al. 2013; Oskay et al. 2020; Jánošíková et al. 2021). In Central Europe, populations of *D. septosporum* have been extensively characterized, with studies reaching different conclusions about their origin and the relative contributions of anthropogenic introductions versus natural distribution (Barnes et al. 2014; Boroń et al. 2021; Drenkhan et al. 2013; Mullett et al. 2015, 2017; Tomšovský et al. 2013). Some of these studies provided evidence supporting a European origin of *D. septosporum* (Barnes et al. 2014; Drenkhan et al. 2013; Mullett et al. 2017). Recent large‐scale analyzes of populations from multiple continents by Mullett et al. (2021) strongly support the Eurasian origin of *D. septosporum*, indicating that Northeastern Europe and Western Asia are likely the center of origin of the pathogen.

The highest genetic diversity of *D. septosporum* populations has been reported from Northeast Europe. These populations exhibit low Nei's genetic distances, low number of clones, high mean allelic and private allelic richness, and high mean haploid genetic diversity, indicating that the fungus may be native to this area (Adamson et al. 2018). These populations of *D. septosporum* are located within the native range of *P. sylvestris* in Europe (Adamson et al. 2018). Previous genetic analyzes of Slovak *D. septosporum* populations also provided evidence of high genetic diversity in populations associated with *P. sylvestris* (Jánošíková et al. 2021).

The occurrence of a pathogen in apparently healthy host tissues may be particularly relevant in systems characterized by long‐term host–pathogen associations. In such systems, the pathogen may occur not only in symptomatic tissues but also in asymptomatic hosts, potentially forming part of the broader microbial community associated with plant tissues (Mesanza et al. 2026). Studying these microbial communities can therefore provide additional insights into the ecological context of host–pathogen association. Detection of *D. septosporum* can be challenging because the pathogen may occur in host tissues even in the absence of visible disease symptoms, for example during early stages of infection. Species‐specific molecular markers, therefore, represent a valuable tool for detecting the pathogen directly from plant tissues, and comparative studies have shown that quantitative PCR (qPCR) provides high diagnostic accuracy for the detection of *D. septosporum* (Ioos et al. 2010; Wartalska et al. 2021).

At the same time, advances in high‐throughput sequencing have enabled detailed investigation of fungal communities associated with plant tissues. Metabarcoding approaches based on amplification of ITS regions allow detection of both dominant and rare fungal taxa and provide insights into the composition of endophytic fungal communities (Peršoh 2013; Millberg et al. 2015; Taudiere et al. 2018; Lazarević and Menkis 2020; Agan et al. 2021). Such approaches can therefore complement species‐specific detection by providing information on the broader fungal community associated with the host.

In this study, we combined species‐specific qPCR detection of *D. septosporum* with ITS metabarcoding of pine needle fungal communities to investigate the occurrence of the pathogen and the composition of fungal communities associated with *P. sylvestris* needles in natural stands in Slovakia. In addition, a pooled non‐natural positive‐reference group with confirmed presence of *D. septosporum* by qPCR was included for comparison. We further assessed differences in fungal community structure among the studied sample groups and investigated fungal taxon co‐occurrence patterns to characterize associations within the needle mycobiota. Classification analysis was used to identify fungal taxa associated with differences among the studied sample groups and to assess whether fungal community composition could distinguish among these groups. We hypothesized that if *D. septosporum* has been present in these ecosystems for a long time, it may occur even in symptomless stands, and its presence may not coincide with major shifts in the overall needle mycobiota. We further hypothesized that the studied sample groups would differ in fungal community structure and co‐occurrence patterns, and that a subset of fungal taxa would contribute to their discrimination.

## 2 Material and Methods

### 2.1 Sampling Sites

Natural stands of Scots pine are not limited to one compact unit in Slovakia. They occur on limestone substrates, are mountainous at an altitude of up to 1156 m on the southwest exposure, the upper limit of sporadic natural occurrence up to 1490 m.a.s.l.

For the experiment stands with native occurrence of *P. sylvestris* located in the north part of Slovakia in Spiš region, in Slovak Paradise National Park (Figure 2; Table 1, Hrabušice and Vernár stands) were selected. Further, old forest stands located in this region were also sampled (Figure 2; Table 1, Danišovce, Spišský Hrhov). We also included a pooled positive‐reference group comprising 15 samples from seven non‐natural stands (i.e., planted or substantially human‐altered *P. sylvestris* stands). The material consisted of archieved needles and DNA extracts collected between 2014 and 2019 from confirmed *D. septosporum* non‐natural *P. sylvestris* stands in Slovakia (Figure 2; Table 2). Hereafter, this pooled category is referred to as the non‐natural positive‐reference group.

**FIGURE 2:**
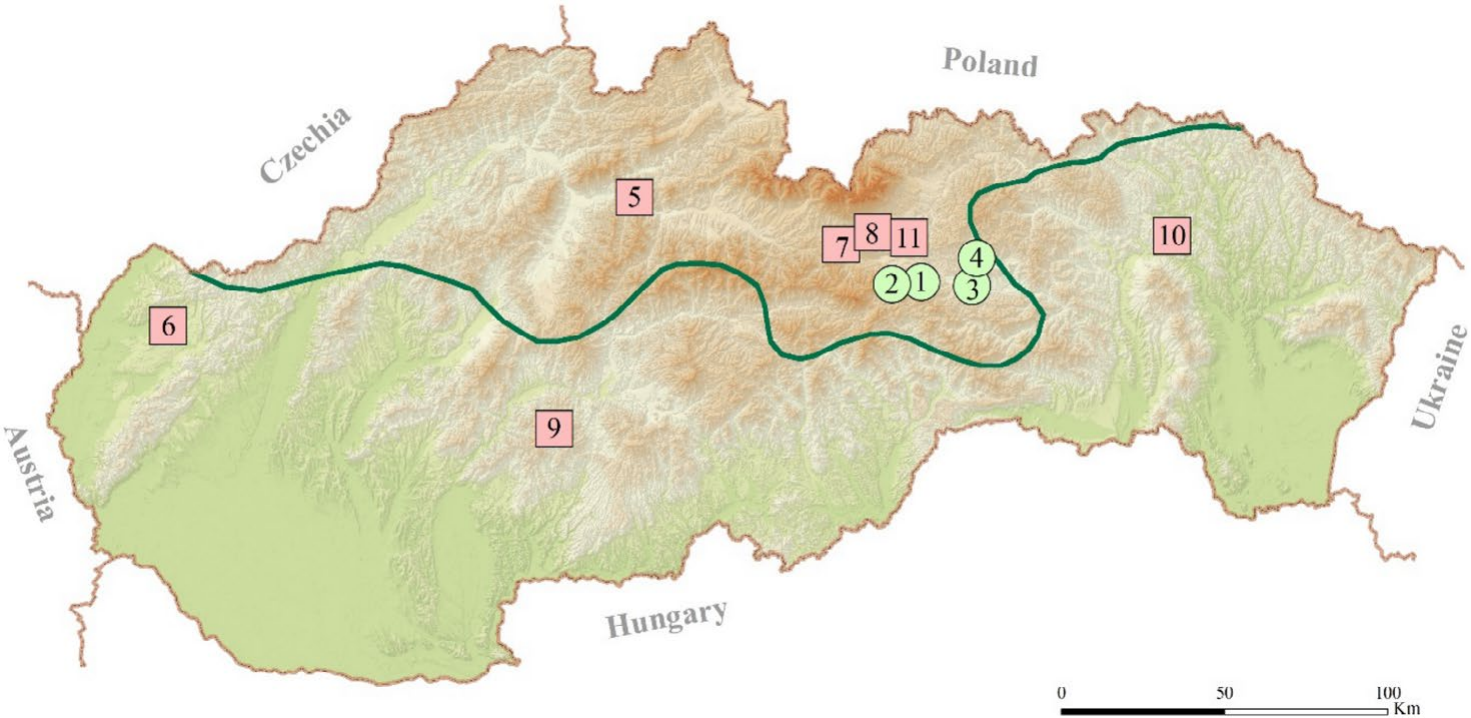
The geographic distribution of *P. sylvestris* stands evaluated in the present study in Slovakia. The green line represents the border of *P. sylvestris* native occurrence. Stands indicated as circle‐shaped signs are experimental stands with native occurrence of *P. sylvestris* , and square ones are non‐natural stands of *P. sylvestris* . Numbers corresponding to stand names are according to Tables 1 and 2.

**TABLE 1:** Characteristics of experimental stands with native occurrence of *Pinus sylvestris* .

Locality	Stand No	Stand specification	GPS coordinates	Altitude (m a.s.l.)	Host age (years)	Slope in %	Exposure	*P. sylvestris* percentage	Nature protection level	Nature conservation zone	Management regime	Number of sampled trees
Slovak Paradise National Park	1	Hrabušice	48.9405430; 20.4059460	600–880	120–180	80–100	S, E, N	35–70	3; 5	100%	Any	13
	2	Vernár	48.9302550; 20.284088	750–920	130	70	SE	40	5	0%	Any	16
Hornád Basin, Forests of the Slovak Republic	3	Danišovce	48.9680820; 20.620381	50–605	135–140	25	NW, SW	87–100	1	0%	Restoration	14
	4	Spišský Hrhov	49.0143730; 20.6317380	490–550	120	35	NE	86	1	0%	Afforest	14

*Note:* Nature protection level indicates the degree of nature protection (1–5), with 1 representing the lowest and 5 the highest degree of protection. Nature conservation zone (%) indicates the proportion of the stand area located within a designated nature conservation zone. Exposure refers to slope aspect; N, NE, E, SE, S, SW, W, and NW indicate north, northeast, east, southeast, south, southwest, west, and northwest, respectively. Management regime refers to the forest management category assigned to the stand in the Slovak Forest Management Information System (ISHLP).

**TABLE 2:** List of samples from non‐natural stands of *P. sylvestris* used in analyzes from previous studies (Jánošíková‐Hečková et al. 2018; Adamčíková et al. 2023).

Locality No.	Locality	No of samples	Date of collection	Source
5	Ľubochňa	1	March 25, 2014	Needles
6	Šajdíkove Humence	3	May 13, 2015	Needle (1)/DNA (2)
7	Sparišská	3	January 29, 2015	Needles
8	Spišská Teplica	4	January 21, 2016, March 13, 2019	Needle (3)/DNA (1)
9	Banská Belá	2	December 4, 2015	DNA
10	Kuková	1	July 10, 2015	DNA
11	Ždiarec	1	January 29, 2015	DNA

*Note:* Source for analyzes: symptomatic needles from herbarium item/directly DNA extract positive for *D. septosporum*.

### 2.2 Sample Collection

Sampling of pine needles was carried out in May 2022. The presence of the DNB pathogen was investigated according to EPPO descriptions of characteristic symptoms such as: needle tip dieback, dead tip of needles in brown/red color, red/brown banding on the needle, and small black fruit bodies in the affected part of the needle. Not only symptomatic needles but also asymptomatic needles were collected. Only fallen needles from the ground under the individual *P. sylvestris* trees were collected due to the height of the trees and the inaccessibility of the crown. Thirteen to sixteen individual trees per stand were sampled. Each sample consisted of at least 50 randomly collected needles beneath each sampled tree, and the sampled trees were spatially separated within the respective stand. Samples were placed in separate bags, sealed and labeled, and stored in a freezer at −20°C until processing. The number of sampled trees per stand is provided in Table 1.

### 2.3 DNA Extraction, Amplification, and Sequencing

In the four natural stands, each sampled tree represented one biological sample. From each tree, a composite needle sample consisting of 3–5 needles was selected and used for a single DNA extraction. Thus, the 57 trees sampled in natural stands (Table 1) resulted in 57 individual DNA extracts. For samples included in the non‐natural positive‐reference group, 8 DNA extracts were obtained from needle samples, while 7 additional DNA extracts obtained in previous studies were used directly for PCR amplification (Table 2). In total, 72 individual DNA samples were included in the molecular analyzes. Each DNA extract was used as a template for PCR amplification, resulting in one individually tagged PCR product per DNA sample. The individual PCR products were subsequently purified, quantified, normalized, and pooled into a sequencing library.

The pine needles were homogenized to powder in a mortar using sand and pestle. DNA was isolated using the GeneJET Plant Genomic DNA Purification Kit (Thermo Scientific, USA) according to the manufacturer's instructions. For fungal community analysis, the ITS2 region was amplified using primers gITS7 (Ihrmark et al. 2012) and ITS4 (White 1990), with all primers containing 6 bp identification sequences (tags). The composition of the PCR mix was as follows: ultrapure H_2_O, Q5 High Fidelity polymerase (New England BioLabs, Ipswich, MA, USA), 10 mM dNTPs, BSA, forward and reverse primers, 1 μL of extracted DNA. Amplification was performed in a Bio‐Rad T100 Thermal Cycler. Initial denaturation (90 s at 98°C) was followed by 35 cycles of denaturation (15 s at 98°C), annealing (15 s at 62°C) and extension (15 s at 72°C). PCR products were purified using AMPureXP magnetic beads (Beckman Coulter Life Science, USA) and quantified on a Qubit fluorometer (Thermo Scientific, Waltham, MA, USA). PCR amplicons were diluted to the same concentration and then pooled. The sequencing library was prepared with the TruSeq LT PCR free kit (Illumina, San Diego, CA, USA) without DNA fragmentation and size selection. The library was quantified by qPCR using the Kapa Illumina Library Quantification kit (Roche, Basel, Switzerland), diluted to 4 nM and denatured prior to sequencing on an Illumina MiSeq instrument with the MiSeq Reagent Kit v3 (600–cycle).

### 2.4 Sequences Analysis

The acquired data were processed in SEED2 (version 2.12) (Vetrovský et al. 2018). Forward and reverse reads were pooled, and all sequences with quality lower than Q30 were discarded. Samples were demultiplexed by tag sequences, and primers were removed. Chimeras were identified and removed using the Vsearch algorithm (Rognes et al. 2016). The obtained sequences were clustered into operational taxonomic units (OTUs) at 97% similarity level, with the most frequent sequence in each cluster identified using the UNITE + INSDC combined dataset (v8.3). Plant ITS region sequences were removed from further analysis. Data were evaluated on the MicrobiomeAnalyst platform (https://www.microbiomeanalyst.ca).

### 2.5 Statistical Analysis

Several statistical analyzes were performed in MicrobiomeAnalyst (https://www.microbiomeanalyst.ca) and R (version 4.2.2) to evaluate the diversity and composition of fungal communities associated with *P. sylvestris* needles. Alpha diversity was assessed using the Chao1 and ACE indices (taxon richness) and the Shannon and Simpson indices (diversity and evenness) to compare the mycobiome among different sample groups. Statistical differences in alpha diversity among groups were tested using the non‐parametric Kruskal–Wallis test, with a statistical significance level set at *p* < 0.05. Beta diversity was assessed based on the Bray–Curtis dissimilarity index and visualized using non‐metric multidimensional scaling (NMDS). The statistical significance of differences in fungal community structure among sample groups was tested using permutation multivariate analysis of variance (PERMANOVA). Pairwise comparisons among groups were further evaluated using PERMANOVA with false discovery rate (FDR) correction to account for multiple testing. Fungal co‐occurrence network analysis was performed at the genus level using the MicrobiomeAnalyst platform with the Sparse Estimation of Correlations among Microbiomes (SECOM, Pearson1) algorithm, using 100 permutations in the “All Groups” analysis mode. Statistically significant correlations (*p* < 0.05) meeting a correlation threshold of |*r*| ≥ 0.3 were retained for network generation. In the resulting network, nodes represented individual fungal genera, with pie charts indicating their relative abundance across the studied sample groups, while edges represented positive or negative associations and edge thickness corresponded to correlation strength. Random Forest (RF) classification was used to identify the taxa contributing most to the differences among groups. RF models were created with 1000 trees, and classification accuracy was assessed using out‐of‐bag (OOB) error as an internal measure of model generalization ability. The number of predictors tested in individual nodes (mtry) was optimized within the range of 2–50. The significance of individual taxa was evaluated based on the degree of decline in classification accuracy after their random permutation.

### 2.6 Real‐Time PCR

The presence of *D. septosporum* in DNA needle samples was assessed using species‐specific quantitative PCR (qPCR). Amplification was performed with the KAPA PROBE FAST qPCR Master Mix (2×) (Roche, Basel, Switzerland) following the manufacturer's recommendations. Each reaction contained qPCR master mix, primers, probe, and 2 μL template DNA in a final reaction volume of 20 μL. Species‐specific primers and a hydrolysis probe targeting *D. septosporum* were used: DStub2‐F1 (5′‐CGAACATGGACTGAGCAAAA‐3′), DStub2‐R1 (5′‐TGCCTTCGTATCTGCATTTC‐3′), and DStub2‐P1 (5′‐ROX‐TGGAATCCACAGACGCGTCA‐BHQ2‐3′) (Ioos et al. 2010). qPCR reactions were carried out on an Agilent MX3005P real‐time PCR system (Agilent Technologies, Santa Clara, CA, USA). The qPCR assay was performed in accordance with the MIQE guidelines (Bustin et al. 2009). Thermal cycling conditions consisted of an initial denaturation step of 3 min at 95°C, followed by repeated cycles of denaturation (3 s at 95°C) and combined annealing/extension steps (20 s at 60°C) optimized for probe‐based detection. Fluorescence signals were recorded during each amplification cycle, and data were analyzed using the instrument's software MxPro QPCR Software v.4.10d. Each run included positive controls containing *D. septosporum* DNA and negative controls (no‐template controls) to monitor amplification specificity and potential contamination. Samples were considered positive when amplification curves crossed the threshold (Ct < 35) within the defined cycle range.

## 3 Results

### 3.1 Presence of DNB Symptoms

Based on visual assessment, among the four *P. sylvestris* stands (Table 1), typical symptoms of DNB were observed only in the Vernár stand. No visible DNB symptoms were recorded in the remaining stands: Hrabušice, Danišovce, and Spišský Hrhov.

### 3.2 Molecular Detection of *D. Septosporum*

The presence of *D. septosporum* was detected across all 72 samples by qPCR using species‐specific primers (Table 3). Individual Ct values and detection statuses are provided in Table S1. The pathogen was detected in the Vernár stand (Slovak Paradise National Park) in 11 out of 16 analyzed samples (68.75%). In the non‐natural positive‐reference group, *D. septosporum* was detected in all analyzed samples (15 out of 15, 100%). In contrast, *D. septosporum* was not detected at the Hrabušice stand (Slovak Paradise National Park), as well as at Danišovce and Spišský Hrhov (Hornád Basin, Forests of the Slovak Republic).

**TABLE 3:** Identification of *Dothistroma septosporum* (DS) in all analyzed samples grouped according to the sample group.

Sample group	Total no. of samples	Symptoms^a^	qPCR of DS positive samples	
			No.	%
Danišovce	14	A	0	0
Hrabušice	13	A	0	0
Non‐natural positive‐reference group	15	P	15	100
Spišský Hrhov	14	A	0	0
Vernár	16	P	11	68.75

^a^ Visual evaluation of typical DNB symptoms presence in the stand on *P. sylvestris* needles: A = absence of symptoms, P = evidence of symptoms‐ red bands and fruiting bodies.

Metabarcoding analysis of ITS sequences showed a high relative abundance of the genus *Dothistroma* in the non‐natural positive‐reference group (27.1%). In the Vernár stand, the genus was present only at low relative abundance (< 1%).

### 3.3 Fungal Community Composition

To provide sample‐level resolution, relative abundance profiles for all 72 individual samples at the class, family, and genus levels are provided in Figures S1–, S3, respectively. The 15 samples included in the non‐natural positive‐reference group are shown individually and labeled according to their seven source localities.

Illumina MiSeq sequencing of the ITS2 region yielded 1,058,455 high‐quality chimera‐free sequences acquired for the ITS2 region. All sequence data were submitted to GenBank databases as part of BioProject PRJNA1455419. The mean number of sequences per sample was 14,701. In total, 1676 OTUs were generated from ITS sequences.

Fungal community structure at the class level (Figure 3) was characterized by consistent dominance of Leotiomycetes in most sample groups, accounting for 71.0%–84.8% of sequences. A clear exception was the non‐natural positive‐reference group, where the relative abundance of Leotiomycetes decreased markedly to 26.0%. In this group, Dothideomycetes represented the most abundant class (41.2%), followed by Tremellomycetes (11.3%) and Sordariomycetes (8.2%). Elevated proportions of Dothideomycetes were also observed in Vernár (10.5%) and Danišovce (8.3%), while Hrabušice showed comparatively lower values (3.6%). Other fungal classes, including Microbotryomycetes (0.6%–3.8%), Eurotiomycetes (2%–6%), and Agaricomycetes (0.7%–9.3%), represented minor components of the needle mycobiome.

**FIGURE 3:**
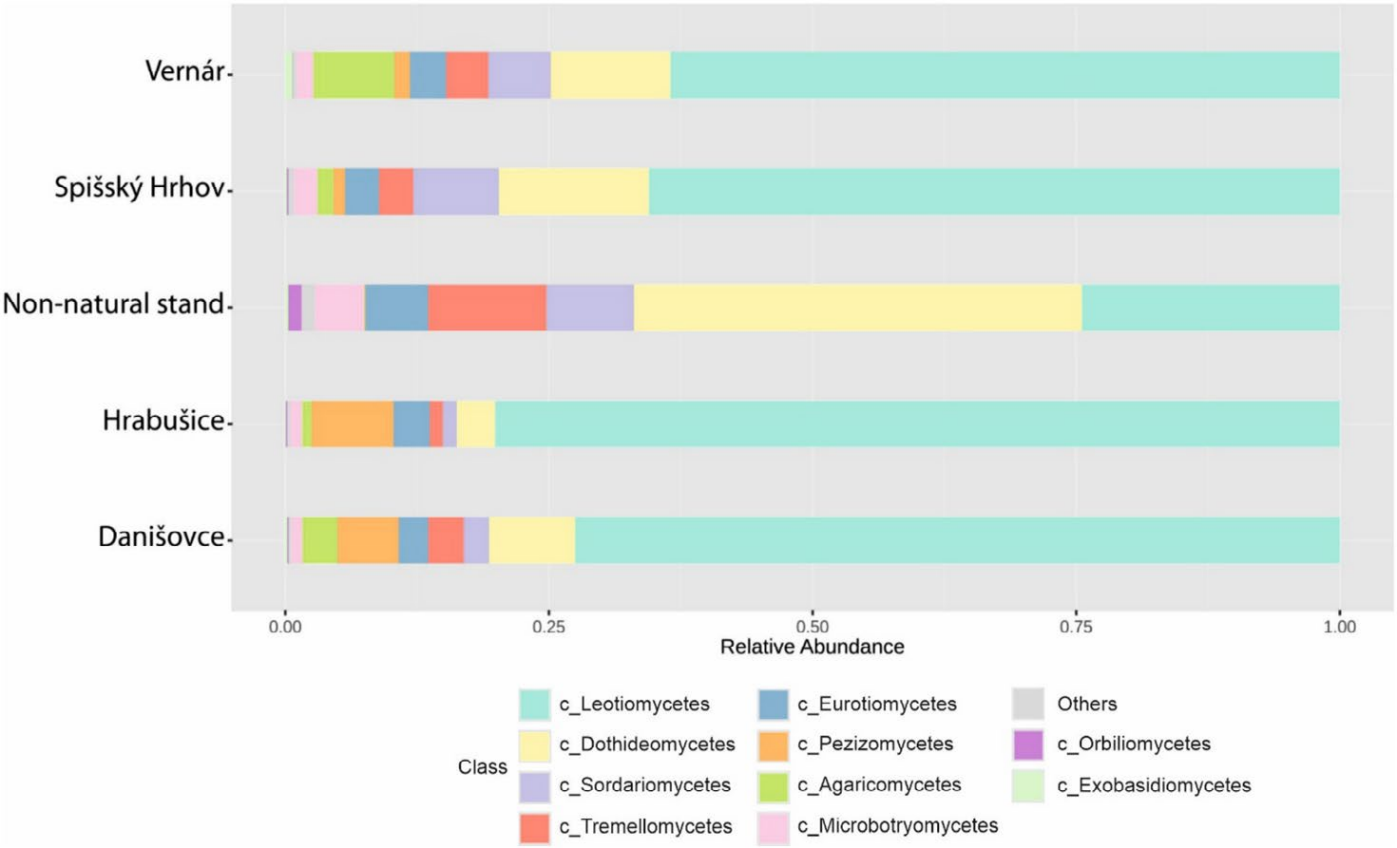
Relative abundance of the 10 most abundant fungal classes. Less abundant taxa are grouped as “Other”. All columns are normalized to 100%. In the figure, “Non‐natural stand” denotes the pooled non‐natural positive‐reference group.

At the family level (Figure 4), *Rhytismataceae* was the most abundant group across all studied sample groups, accounting for 26%–55% of the total sequences. The highest relative abundances were recorded in Hrabušice (54.7%) and Vernár (36.7%), followed by Danišovce (42.7%) and Spišský Hrhov (40.6%). In contrast, the non‐natural positive‐reference group exhibited a markedly different pattern: *Rhytismataceae* accounted for just 17.1% of sequences, whereas *Mycosphaerellaceae* was highly dominant at 27.2%, reflecting the high representation of the genus *Dothistroma*.

**FIGURE 4:**
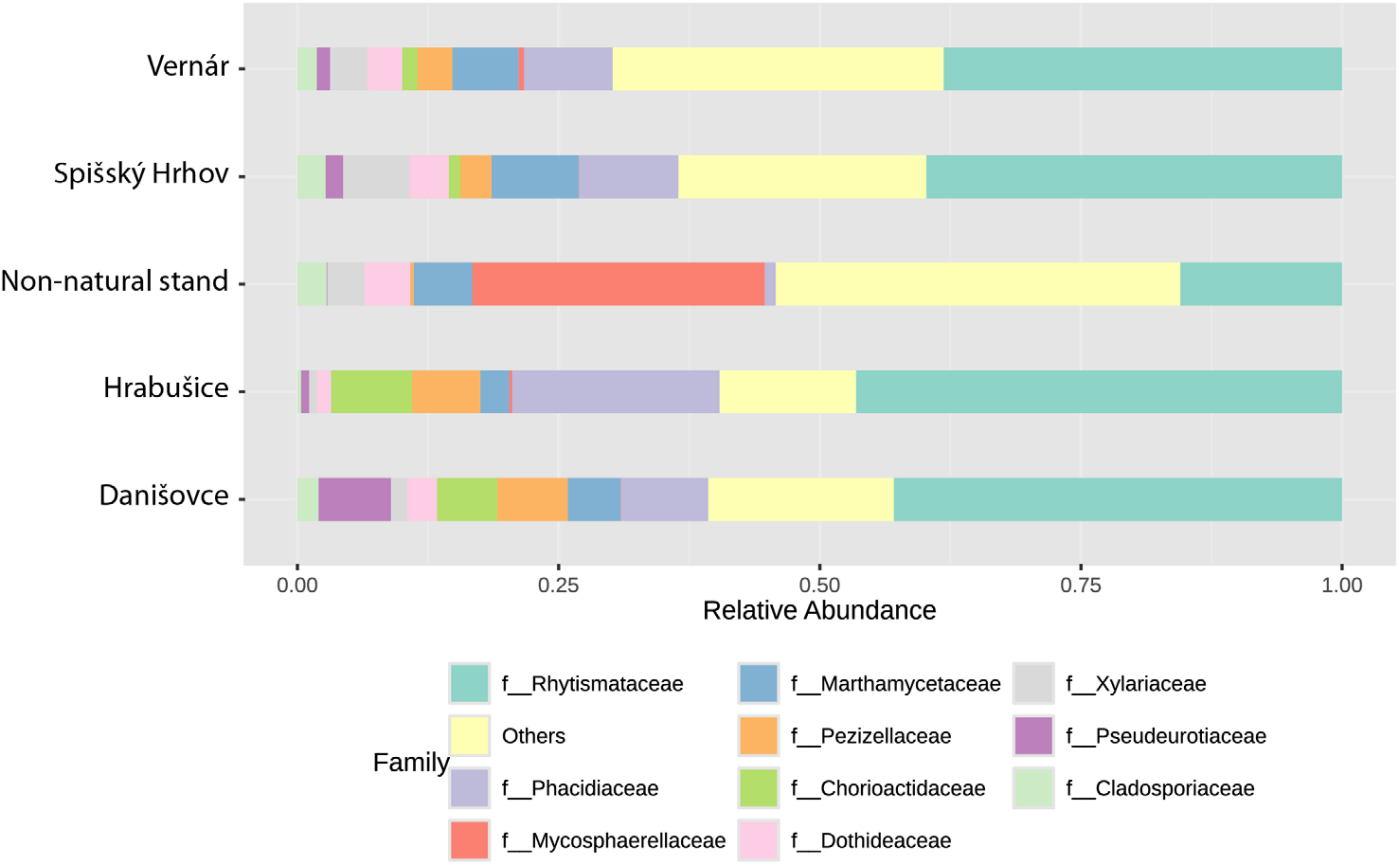
Relative abundance of the 10 most abundant fungal families. Less abundant taxa are grouped as “Other”. All columns are normalized to 100%. In the figure, “Non‐natural stand” denotes the pooled non‐natural positive‐reference group.

Analysis of genus‐level composition (Figure 5) revealed that *Lophodermium* was the most abundant, with relative abundances ranging from 17.2% to 54.8% across all sites. The genus *Phacidium* was most abundant in Hrabušice (17.3%), with lower proportions observed at the other sites (1.0%–9.7%). The genus *Cyclaneusma* was present in all localities, representing 2.5%–8.4% of the fungal community. The genus *Dothistroma* was highly abundant exclusively in the non‐natural positive‐reference group, where it accounted for 27.1% of all sequences. In the Vernár stand, the genus was present only at low relative abundances (< 1%).

**FIGURE 5:**
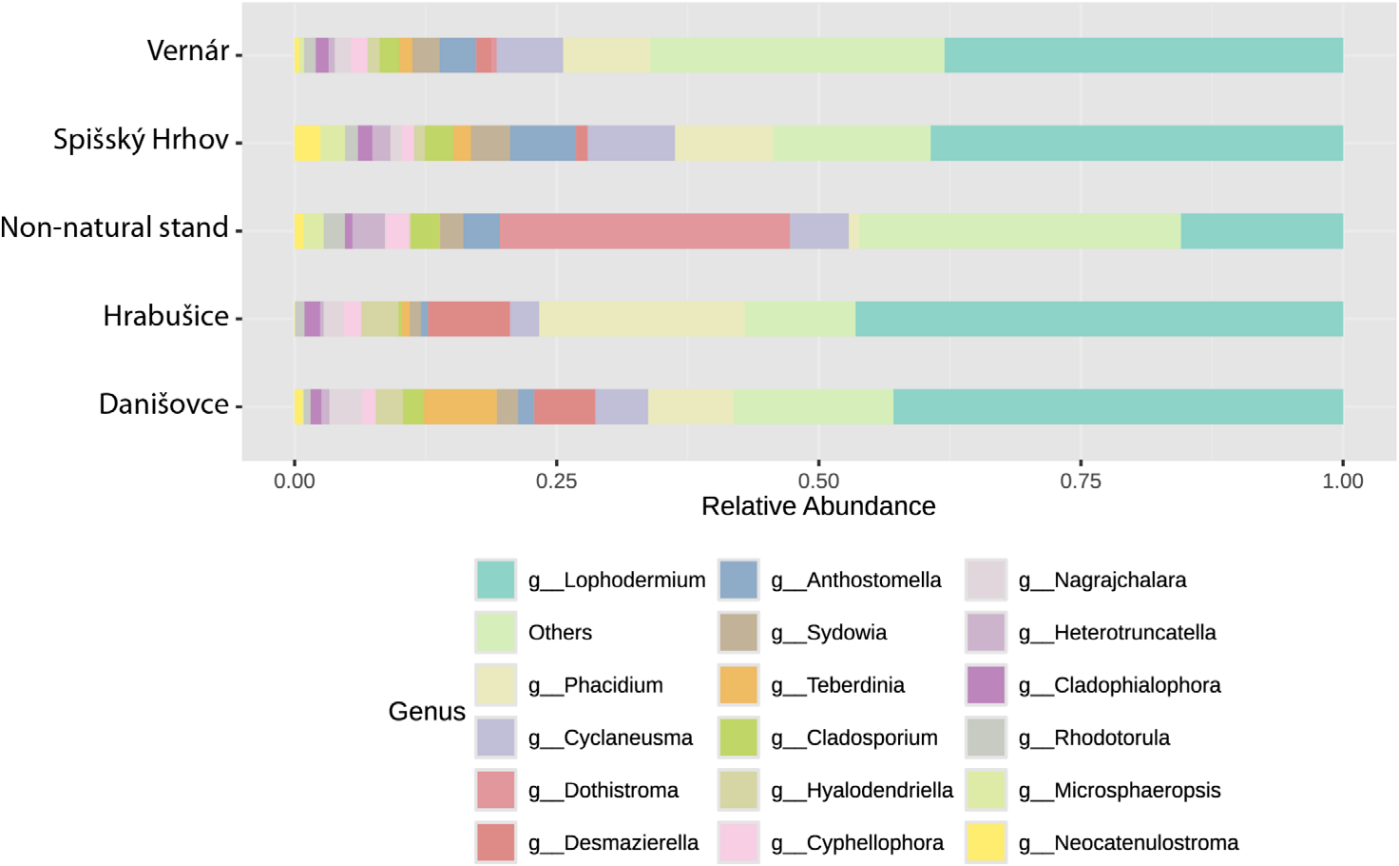
Relative abundance of 17 fungal genera. Less abundant taxa are grouped as “Other”. All columns are normalized to 100%. In the figure, “Non‐natural stand” denotes the pooled non‐natural positive‐reference group.

### 3.4 Co‐Occurrence Patterns of Fungal Genera

The co‐occurrence network consisted of approximately 30 nodes and 80 edges, indicating a moderately connected structure. The majority of associations were positive (approximately 85%–90%), while negative associations were less frequent. The network was organized into several clusters, with no single dominant component (Figure 6). Correlation coefficients ranged from moderate to strong (*r* = 0.52–0.76), indicating structured relationships within the fungal community. Among the strongest positive associations were those involving *Dothistroma*, particularly with *Genolevuria* (*r* = 0.755) and *Alternaria* (*r* = 0.598). Similarly, strong positive correlations were observed between *Fusarium* and *Penicillium* (*r* = 0.722), and among yeast‐like taxa such as *Sporobolomyces*, *Piskurozyma*, and *Krasilnikovozyma* (*r* = 0.54–0.69), suggesting the presence of co‐occurring clusters within the community. In contrast, several notable negative correlations were detected, including associations between *Dothistroma* and *Lophodermium* (*r* = −0.639) and *Phacidium* (*r* = −0.628), indicating potential mutual exclusion patterns among dominant needle‐associated taxa. Negative associations were also observed between *Extremus* and *Cyclaneusma* (*r* = −0.556), as well as between *Neocatenulostroma* and *Lachnellula* (*r* = −0.530). Overall, the network structure suggests the presence of both positive and negative relationships among fungal taxa. Several genera, including *Dothistroma*, *Alternaria*, *Genolevuria*, *Fusarium*, and *Penicillium*, showed a high number of significant associations, suggesting a potentially important role in structuring the network.

**FIGURE 6:**
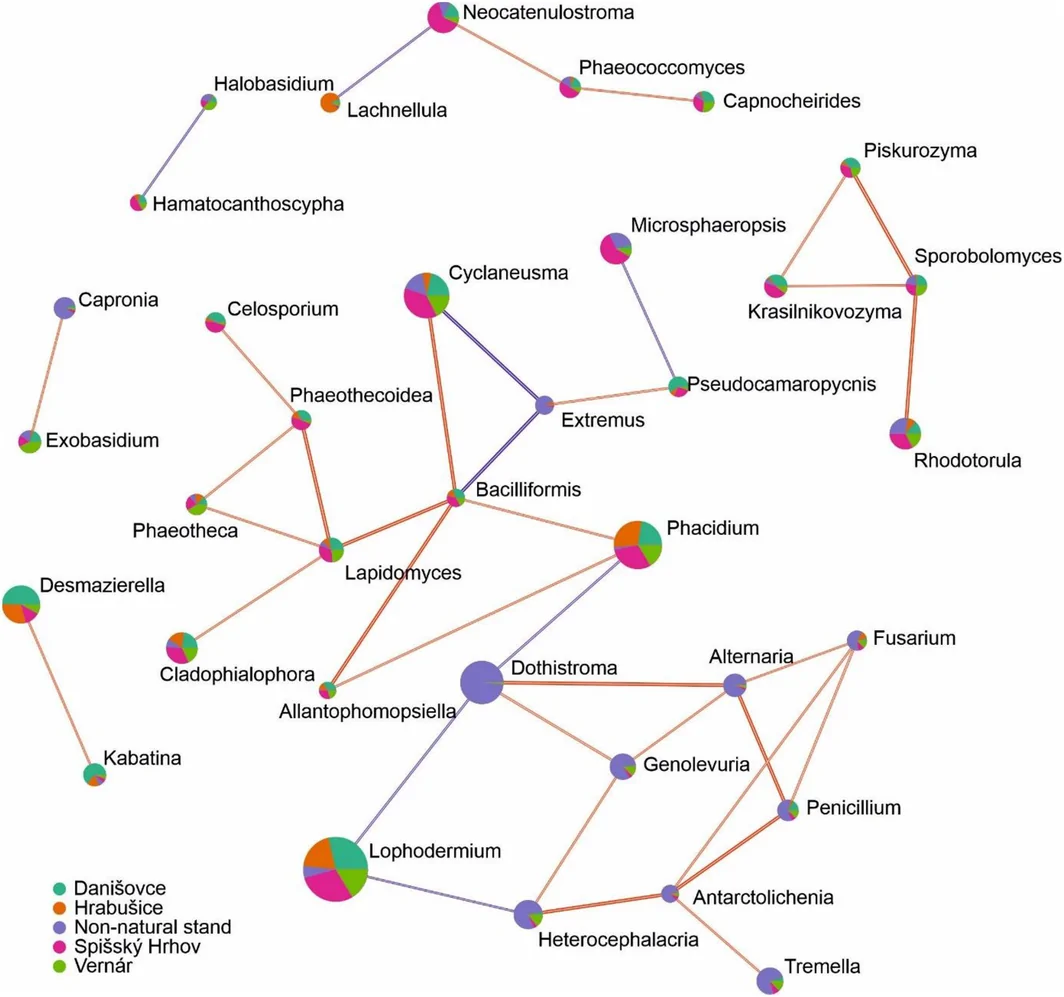
Co‐occurrence network of fungal genera associated with *Pinus sylvestris* needles. Nodes represent genera and edges indicate significant correlations (*p* < 0.05). Edge color shows positive (red) and negative (blue) associations, and edge thickness reflects correlation strength. In the figure, “Non‐natural stand” denotes the pooled non‐natural positive‐reference group.

### 3.5 Alpha Diversity of Fungal Communities

All calculated alpha diversity indices (Chao1, ACE, Shannon, and Simpson) (Figure 7) showed consistent patterns across the studied sample groups. Richness estimators Chao1 (Kruskal–Wallis, *p* < 0.001) and ACE (Kruskal–Wallis, *p* < 0.001) indicated the highest fungal richness in Spišský Hrhov. High but more variable richness was also observed in Danišovce. In contrast, the Hrabušice stand and the non‐natural positive‐reference group consistently exhibited lower richness values. The Vernár stand showed intermediate richness with moderate variability among samples.

**FIGURE 7:**
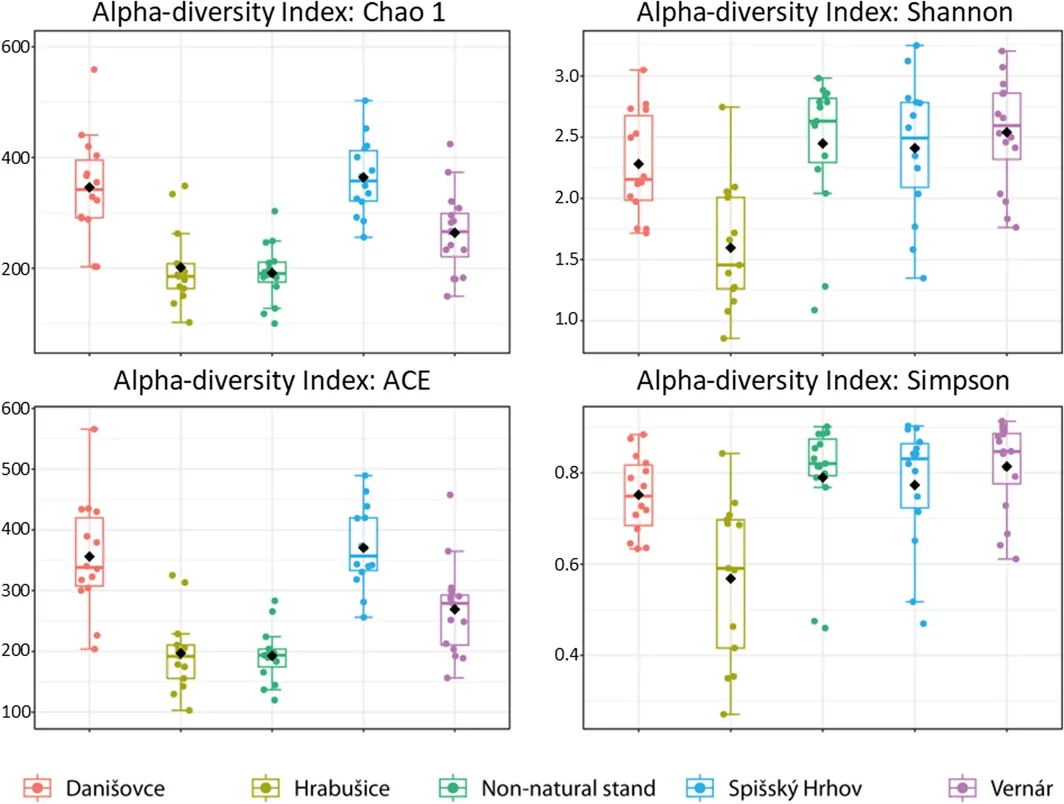
Alpha diversity indices (Chao1, ACE, Shannon, Simpson) showing differences across sample groups. In the figure, “Non‐natural stand” denotes the pooled non‐natural positive‐reference group.

Indices reflecting both richness and evenness Shannon (Kruskal–Wallis, *p* < 0.001) and Simpson (Kruskal–Wallis, *p* < 0.001) indicated generally high diversity across most sample groups. However, the Hrabušice stand displayed pronounced variability, including several samples with markedly reduced diversity, indicating uneven community structure. In contrast, the non‐natural positive‐reference group showed relatively stable and high Simpson values, suggesting more even communities despite lower richness.

### 3.6 Beta Diversity Among Sample Groups

Beta diversity analysis based on Bray–Curtis dissimilarity showed significant differences in fungal community composition among sample groups (PERMANOVA: *F* = 6.6739, *R* ^2^ = 0.287, *p* = 0.001) (Figure 8). The strongest differences were observed between Hrabušice and the non‐natural positive‐reference group (*R* ^2^ = 0.339, *p* = 0.0017), and between Danišovce and the non‐natural positive‐reference group (*R* ^2^ = 0.313, *p* = 0.0017), indicating substantial shifts in fungal community composition.

**FIGURE 8:**
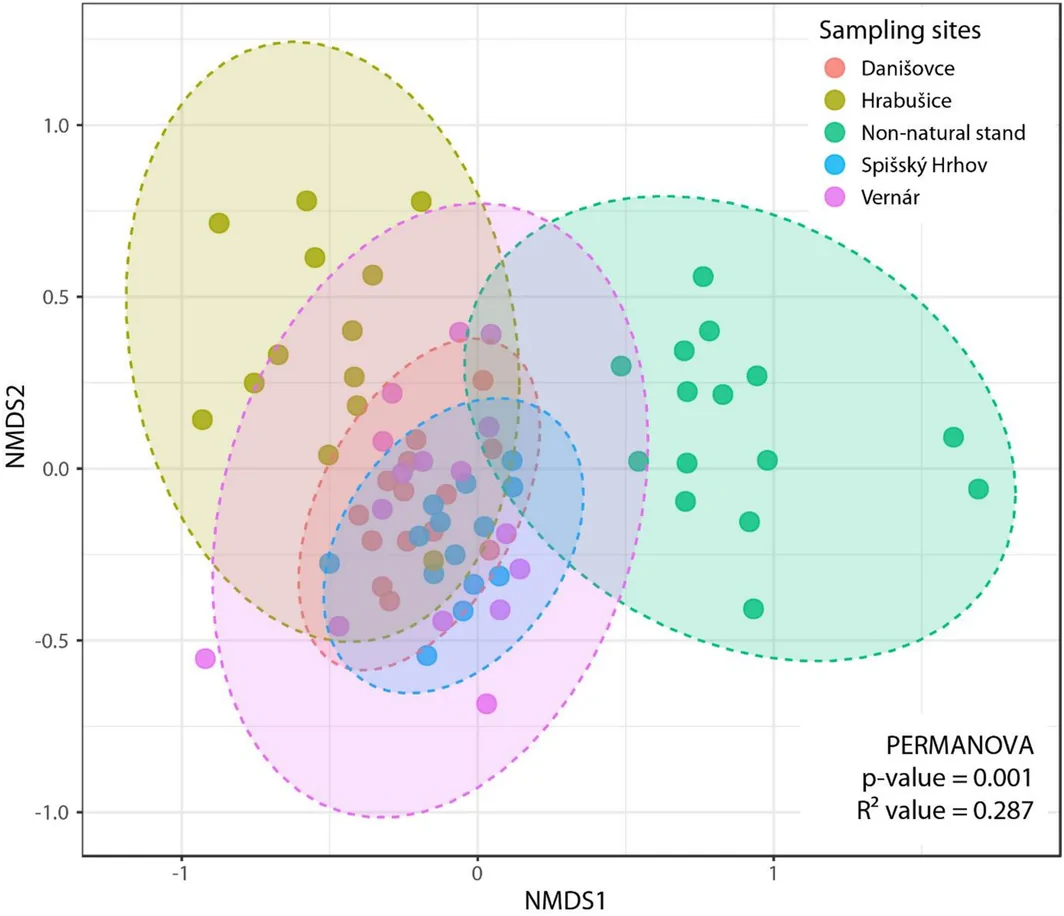
Beta diversity. Non‐metric multidimensional scaling analysis (NMDS) representing the variation in the fungal communities between the five different sample groups (PERMANOVA, *F* = 6.739; *R* ^2^ = 0.287; *p* < 0.001). In the figure, “Non‐natural stand” denotes the pooled non‐natural positive‐reference group.

Significant differences were also detected between Hrabušice and both Spišský Hrhov (*R* ^2^ = 0.124, *p* = 0.0017) and Vernár (*R* ^2^ = 0.137, *p* = 0.0017), as well as between the non‐natural positive‐reference group and Spišský Hrhov (*R* ^2^ = 0.268, *p* = 0.0017) and Vernár (*R* ^2^ = 0.235, *p* = 0.0017). Danišovce also differed significantly from Vernár (*R* ^2^ = 0.084, *p* = 0.0029) and Hrabušice (*R* ^2^ = 0.086, *p* = 0.0125).

In contrast, no significant difference was observed between Danišovce and Spišský Hrhov (*p* = 0.067), while the difference between Spišský Hrhov and Vernár, although statistically significant (*R* ^2^ = 0.063, *p* = 0.031), showed only a weak effect size.

### 3.7 Random Forest Classification of Fungal Communities

Random Forest classification was used to discriminate among fungal communities from the studied sample groups. The model achieved an overall out‐of‐bag (OOB) error rate of 0.111 (Figure 9), indicating a relatively good classification performance.

**FIGURE 9:**
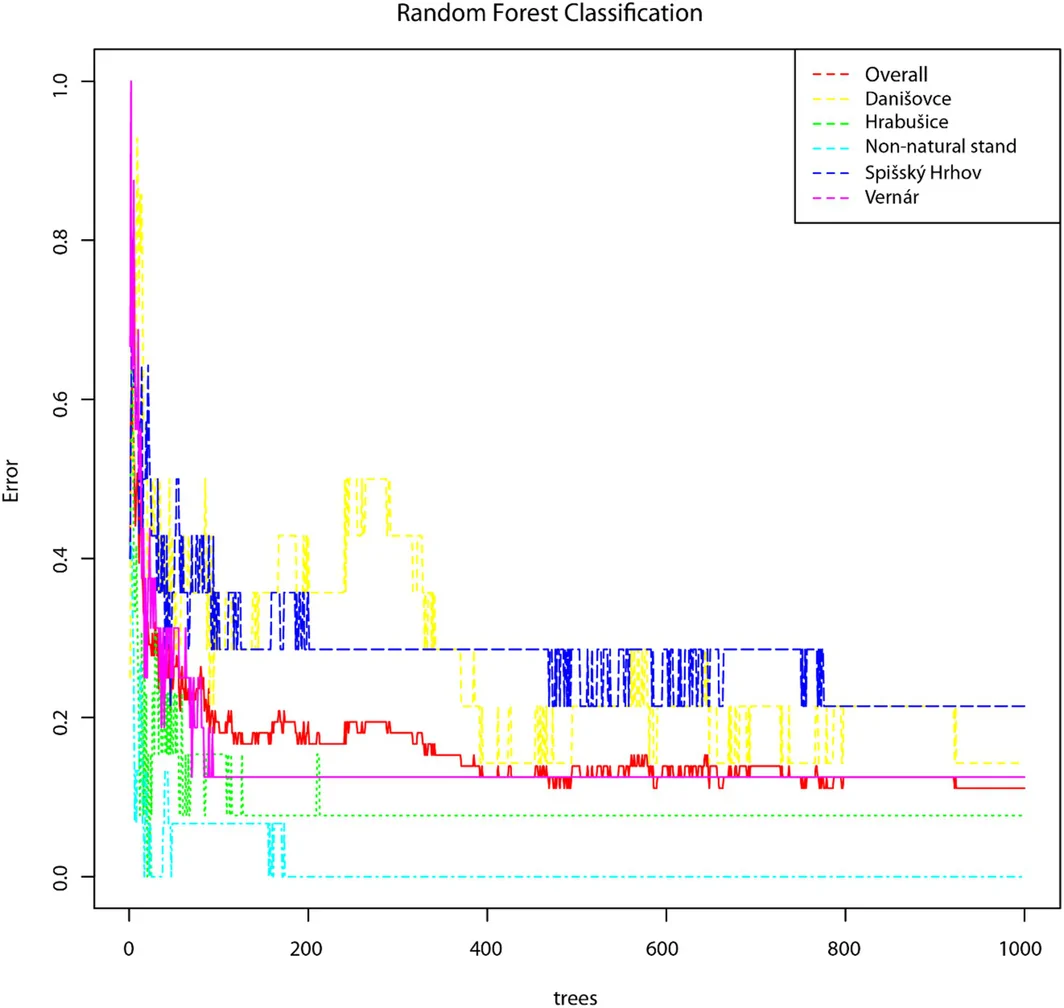
Random Forest classification error rates as a function of the number of trees in the model. The plot shows overall model error and class‐specific error rates for individual sample groups. “Non‐natural stand” denotes the pooled non‐natural positive‐reference group.

Classification accuracy varied among sample groups (Table 4). The highest accuracy was observed for the non‐natural positive‐reference group, which was classified without error (class error = 0.000). Relatively low error rates were also observed for Hrabušice (0.0769) and Vernár (0.125), while higher misclassification occurred in Danišovce (0.143) and Spišský Hrhov (0.214). Misclassifications were primarily observed between natural stands.

**TABLE 4:** Confusion matrix showing classification accuracy for samples across individual sample groups.

	Danišovce	Hrabušice	Non‐natural positive‐reference group	Spišský Hrhov	Vernár	class.error
Danišovce	12	0	0.0	1.0	1.0	0.143
Hrabušice	1	12	0.0	0.0	0.0	0.0769
Non‐natural positive‐reference group	0	0	15.0	0.0	0.0	0.0
Spišský Hrhov	2	0	0.0	11.0	1.0	0.214
Vernár	2	0	0.0	0.0	14.0	0.125

*Note:* Values represent the number of samples classified into each category; class.error indicates the proportion of misclassified samples.

The most important features contributing to classification were identified using Mean Decrease Accuracy (MDA) (Figure 10). The highest contribution was observed for *Dothistroma* OTU0004, followed by several OTUs affiliated with *Lophodermium* (e.g., OTU0022, OTU0144, OTU0129, OTU0081, OTU0141), and *Neocatenulostroma* (e.g., OTU0052, OTU0247, OTU0010, OTU0278). Additional important taxa included *Genolevuria* OTU0070, *Vishniacozyma* OTU0024, *Heterocephalacria* OTU0023, and *Saxophila* OTU0148.

**FIGURE 10:**
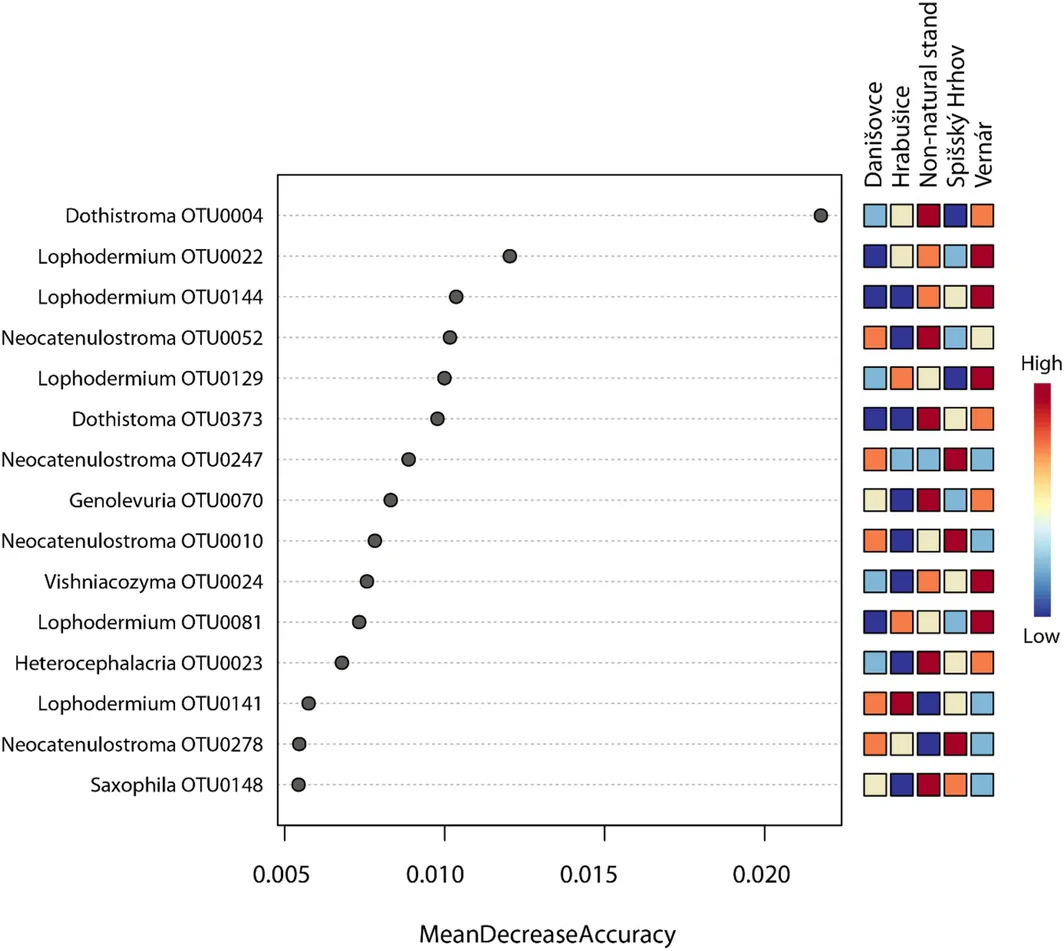
Top OTUs contributing to Random Forest classification based on Mean Decrease Accuracy (MDA). The heatmap shows relative abundance patterns of these OTUs across the studied sample group. In the figure, “Non‐natural stand” denotes the pooled non‐natural positive‐reference group.

## 4 Discussion

### 4.1 Molecular Detection of *D. septosporum* in *P. sylvestris* Stands

This study investigated the occurrence of *D. septosporum* and the composition of fungal communities associated with *P. sylvestris* needles in natural stands in Slovakia. The pathogen was detected using species‐specific qPCR, and the associated fungal communities were analyzed using ITS metabarcoding. Together, these approaches allowed us to assess whether the presence of *D. septosporum* is associated with changes in the overall needle mycobiota.

Previous genetic study of the Slovak *D. septosporum* population based on microsatellite markers showed that the population colonizing *P. sylvestris* had the highest gene diversity despite having the lowest number of isolates. The population also exhibited the lowest clonal fraction, pointing out that most genotypes were genetically distinct (Jánošíková et al. 2021). These findings, together with the indigenous status of *P. sylvestris* in Slovakia and the generally low incidence and severity of DNB in native stands, where typical disease symptoms are often absent (Jánošíková‐Hečková et al. 2018; Jánošíková et al. 2021), are consistent with the possibility of a long‐term association between the host and pathogen. Such patterns have been described for host–pathogen systems characterized by prolonged coexistence and reciprocal evolutionary processes (Ennos 2001; Harrington and Wingfield 1998). In the present study, however, *D. septosporum* was detected in only one of the sampled natural stands. The pathogen was detected at a relatively high frequency (> 68%) in samples from the Vernár stand, where typical DNB symptoms were also observed. Several characteristics of the Vernár stand may be relevant to the observed occurrence of *D. septosporum*. This stand was located closest to a regularly used, busy road and human settlement, potentially increasing opportunities for anthropogenic introduction or dispersal of the pathogen. Human‐mediated movement of infected plant material has been identified as an important pathway for the long‐distance spread of *D. septosporum*, and population‐genetic studies have provided evidence for anthropogenic introductions and subsequent spread of the pathogen (Barnes et al. 2014; Boroń et al. 2021). However, *D. septosporum* is also capable of natural dispersal over considerable distances (Mullett et al. 2016), and the relative contribution of anthropogenic versus natural dispersal cannot be determined from the present data. The presence of *P. nigra* in the stand may also be relevant, as this species has been reported as susceptible to DNB in Slovakia (Jánošíková‐Hečková et al. 2018; Ondrušková et al. 2020), and DNB symptoms were observed on *P. nigra* needles at this site (unpublished data). The stand was also outside a nature conservation zone. These characteristics may represent conditions favorable for pathogen occurrence, although the relative contribution of individual factors cannot be determined from the present study.

Northeastern Europe is considered the most probable center of origin of the pathogen (Mullett et al. 2021), which corresponds to the core of the native range of *P. sylvestris* , the sole native pine species in this region (Adamson et al. 2018). Furthermore, the similarity in fungal community between DNB‐symptomatic and DNB‐asymptomatic *P. sylvestris* needles from this part of Europe supports the long‐term co‐evolution of *D. septosporum* with *P. sylvestris* and its associated mycobiota. *Dothistroma septosporum* can be ecologically integrated into the needle‐associated fungal community rather than causing strong disruptions at the local microbial scale (Agan et al. 2021). Perry et al. (2016) provided evidence for the evolution of reduced susceptibility in Scots pine, potentially in response to the presence of high historical levels of *D. septosporum*. This suggests that the pathogen has been an integral component of the native Caledonian pinewood ecosystem for an extended period. In natural forest stands, *D. septosporum* was also detected in *P. sylvestris* samples collected from southern Europe, specifically in Slovenia, where *P. sylvestris* is native and can be part of the natural forest structure (Piškur et al. 2013; Piškur, personal communication). The present study provides only limited support for widespread persistence of the pathogen in natural Slovak stands. In particular, *D. septosporum* was not detected in the other three natural stands examined, and its detection in Vernár was associated with visible DNB symptoms rather than with an asymptomatic infection state.

No detailed studies have been published on the possibility of *Dothistroma* as an endophyte. The first study (Wartalska et al. 2021), which examined samples for the presence of *D. septosporum* in asymptomatic needles of healthy *P. sylvestris* trees, did not provide evidence for endophytism in *Dothistroma*. The present results also provide no clear evidence that *D. septosporum* commonly occurs as a latent endophyte in symptomless *P. sylvestris* needles. The present findings therefore suggest that the detectable occurrence of *D. septosporum* in the sampled natural stands was uncommon and, where detected at higher frequency, was associated with symptomatic needles rather than providing evidence for widespread asymptomatic or latent colonization.

### 4.2 Fungal Community Composition and Diversity

The ITS metabarcoding results demonstrated significant differences in fungal community composition among the studied sample groups. Although alpha‐diversity measures indicated generally high fungal diversity across the stands, Bray–Curtis‐based beta‐diversity analysis revealed significant differences in community composition. The significant differences observed among groups support the hypothesis that the studied sample groups differ in their fungal community structure.

At the taxonomic level, the fungal communities were dominated by taxa commonly associated with conifer needles, particularly members of *Rhytismataceae* and the genus *Lophodermium*. Similarly, in other studies, species of the genus *Lophodermium* were found to be the most abundant and commonly detected fungal species in needles of *P. sylvestris* in Estonia, Norway (Agan et al. 2021), Sweden (Millberg et al. 2015), and Scotland (Schönrogge et al. 2022). The relatively consistent occurrence of *Lophodermium* across the natural stands contrasts with the markedly different composition observed in the non‐natural positive‐reference group, where *Dothideomycetes* and *Mycosphaerellaceae* were more strongly represented and *Dothistroma* reached substantially higher abundance. These differences suggest that the fungal assemblage of the non‐natural positive‐reference group was not simply a more or less diverse version of the natural communities, but represented a distinct community configuration. In the non‐natural positive‐reference group, the two most abundant genera were *Dothistroma* and *Lophodermium*. The relative abundance of the two frequently detected genera, *Lophodermium* and *Phacidium*, was significantly lower compared to the abundance in the *Dothistroma*‐negative natural stands (Hrabušice, Danišovce, Spišský Hrhov). These results indicate an association between *Dothistroma* occurrence and differences in the relative abundance of these genera; however, the effects of pathogen presence cannot be separated from differences in stand type, locality, sampling period, and sample history in the present dataset. The relatively small differences in alpha diversity compared with the stronger differences in beta diversity are also noteworthy. This pattern indicates that the stands may contain broadly comparable levels of fungal richness and diversity while differing considerably in taxonomic composition. Thus, the main distinction among stands appears to involve the identity and relative abundance of fungal taxa rather than a general loss or gain of fungal diversity. This is particularly relevant to the interpretation of *D. septosporum* occurrence, because the presence of the pathogen in Vernár did not coincide with a pronounced reduction in overall fungal diversity. Instead, its occurrence was embedded within a diverse fungal assemblage.

In our study, Hrabušice, the stand with the highest level of protection, located within a nature conservation zone without management, formed a separate cluster and showed the strongest compositional differences from the non‐natural positive‐reference group. Although these differences may be consistent with an effect of management or conservation status, the present design does not allow these factors to be separated from locality and other stand characteristics. In contrast, old forest stands in Danišovce and Spišský Hrhov, as well as the unmanaged stand with native occurrence of *P. sylvestris* in Vernár, showed a similar community structure. These patterns suggest that forest management, stand history, and local environmental conditions may be associated with variation in fungal community composition in pine needles. However, the individual contribution of these factors cannot be disentangled in the present study.

### 4.3 Co‐Occurrence Patterns Within the Needle Mycobiota

The co‐occurrence network provided a complementary perspective on the organization of the fungal communities by examining associations among individual taxa. It was dominated by positive associations, suggesting that many fungal taxa share similar ecological niches or respond similarly to environmental filtering (Faust and Raes 2012). The presence of several moderately connected clusters supports the view of a structured, but not highly integrated, needle‐associated mycobiome.

*Dothistroma* was integrated within the network and associated with multiple co‐occurring genera; however, these patterns primarily reflect the non‐natural positive‐reference group where the pathogen was present. This indicates that when present, its occurrence is linked to the existing fungal community rather than forming an isolated component. For *Dothistroma*, strong positive associations were observed with *Genolevuria* and *Alternaria*, whereas strong negative associations were shown with *Lophodermium* and *Phacidium*. The latter relationships are particularly noteworthy because *Lophodermium* and *Phacidium* were important components of the Scots pine needle fungal communities. These negative associations may reflect differences in ecological niches or potential indirect competitive relationships among dominant taxa (Oono et al. 2015). Nevertheless, as cautioned by Faust and Raes (2012), these co‐occurrence patterns must be interpreted as statistical correlations that require further experimental validation to confirm direct biological interactions.

The network structure was consistent with the community‐level patterns revealed by beta diversity results, showing that differences among sample groups were accompanied not only by variation in overall fungal composition but also by differences in associations among individual taxa. In particular, the contrast between the non‐natural positive reference group, where *Dothistroma* was highly abundant, and Vernár, where the pathogen was detected by qPCR but represented < 1% of the fungal community, indicates that its occurrence was associated with a distinct fungal community context. Similar co‐occurrence patterns and network structures have been reported in other host‐associated microbial communities (Poudel et al. 2016). In our study, these patterns suggest that, despite differences in community composition among the sampled stands, some taxa tended to co‐occur within the dataset. Overall, the network analysis therefore complements the community‐level results by suggesting that *Dothistroma* occurrence was embedded within the broader structure of the needle mycobiota rather than occurring independently of the surrounding fungal community.

### 4.4 Random Forest Classification and Discriminatory Taxa

Random Forest classification further addressed whether the observed differences in fungal community composition were sufficiently consistent to discriminate among the studied sample groups and which fungal taxa contributed most to this discrimination. The relatively low overall out‐of‐bag error rate (0.111) indicates good classification performance. The non‐natural positive‐reference group was classified without error, whereas misclassification occurred mainly among the natural stands. This pattern suggests that the non‐natural positive‐reference group had a particularly distinct fungal community profile, while the natural stands retained greater compositional overlap despite the significant differences detected by beta‐diversity analysis. The use of out‐of‐bag observations provides an internal estimate of classification error and is a standard feature of Random Forest models (Breiman 2001).

The complete classification of the non‐natural positive‐reference group is consistent with its distinctive fungal composition and with the high prevalence and abundance of *Dothistroma* in this actively infected reference system. In contrast, the greater overlap among natural stands suggests that their differentiation was not determined by a single taxon but by variation in the relative abundance of multiple fungal taxa. The Random Forest results therefore provide additional support for the hypothesis that fungal community structure differs among sample groups while indicating that the magnitude and nature of this differentiation vary among groups.

MDA identified *Dothistroma* OTU0004 as the most important discriminatory feature, followed by several OTUs affiliated with *Lophodermium* and *Neocatenulostroma*. In Random Forest analysis, permutation‐based variable importance is assessed from the decline in classification accuracy following random permutation of individual predictors, thereby identifying variables that contribute to the predictive discrimination of groups (Breiman 2001). The high importance of *Dothistroma* is particularly relevant to the main objective of the study, as it indicates that the distribution of this taxon contributed substantially to distinguishing the actively infected non‐natural positive‐reference group from the natural stands. However, the contribution of multiple *Lophodermium* and *Neocatenulostroma* OTUs demonstrates that discrimination was not driven by *Dothistroma* alone, but reflected differences involving several components of the needle mycobiota. Importantly, MDA identifies features that contribute to classification and should not be interpreted as evidence that these taxa causally determine community structure.

### 4.5 Study Limitations

Several limitations should be considered when interpreting the observed differences in fungal community composition. The natural stands were sampled during a single field season, whereas the non‐natural comparison material included archived samples collected from several localities and years and selected based on confirmed *D. septosporum* positivity. Consequently, stand type, locality, sampling period, pathogen status, and sample history are not independent in the present dataset, and their individual effects cannot be disentangled. The observed differences in fungal community composition should therefore be interpreted as associations among the sampled groups rather than as evidence of a causal effect of stand type, pathogen occurrence, or any individual environmental factor.

Samples collected from the natural stands consisted of naturally fallen needles, in which fungal communities may change after senescence through decomposition and secondary colonization. Previous studies of *P. sylvestris* needles have demonstrated temporal changes in fungal communities during needle senescence and after needle fall, including succession of fungal taxa during decomposition (Tokumasu 1998; Tokumasu et al. 1994; Virzo De Santo et al. 2002). Consequently, taxa detected in these samples cannot always be distinguished as fungi established in living needles from those that colonized the needles after abscission. Sampling living foliage was not feasible because of the age and height of the trees studied. The results from the natural stands should therefore be interpreted as characterizing fungal communities associated with fallen *P. sylvestris* needles rather than as a direct representation of the mycobiome of living foliage.

## 5 Conclusion

This study combined species‐specific qPCR and ITS metabarcoding to assess the occurrence of *D. septosporum* and its relationship with needle‐associated fungal communities of *P. sylvestris* in Slovakia. The pathogen was detected only in one natural stand with visible disease symptoms, while it was not detected in other sampled native stands without symptoms. In contrast, it was consistently detected and highly abundant in the non‐natural positive‐reference group. These findings do not support the assumption that *D. septosporum* is a regular, cryptic component of needle mycobiomes in native Scots pine ecosystems in this region. Instead, high abundance was observed in the pooled non‐natural positive‐reference group, while the present design does not allow this pattern to be attributed specifically to stand type.

Fungal communities in natural stands were relatively consistent and dominated by typical needle‐associated taxa, members of the *Ascomycota* phylum, with *Lophodermium* as the most abundant genus across all sites. Whereas the non‐natural positive‐reference group showed marked shifts in community composition and structure, including increased abundance of *Dothideomycetes* and *Dothistroma*. Co‐occurrence patterns further indicated both potential competitive and facilitative interactions, suggesting that the establishment and proliferation of *D. septosporum* may be influenced by broader community dynamics.

Overall, our results highlight that pathogen detection using eDNA approaches needs to be interpreted within an ecological context. The combination of targeted qPCR and community‐level metabarcoding provides a useful framework for examining pathogen occurrence together with the broader fungal community. However, the observed patterns should be considered in light of the limitations of fallen‐needle sampling and the non‐harmonized sampling history of the comparison material. Future studies based on standardized sampling of a larger number of fresh needles from trees, including asymptomatic tissues, replicated across seasons and localities, will be important for determining whether *D. septosporum* occurs consistently at low abundance in symptomless native Scots pine stands. The use of complementary species‐specific assays targeting multiple genomic regions could further strengthen pathogen detection. Such approaches would also help disentangle pathogen‐associated effects from environmental and sampling‐related variation.

## Author Contributions

**Renata Artimová:** formal analysis (equal), investigation (equal), methodology (equal), writing – original draft (equal), writing – review and editing (equal). **Katarína Adamčíková:** conceptualization (equal), investigation (equal), methodology (equal), supervision (equal), writing – original draft (equal), writing – review and editing (equal). **Juraj Medo:** data curation (equal), formal analysis (equal), investigation (equal), validation (equal), writing – review and editing (equal). **Marek Kobza:** formal analysis (equal), visualization (equal), writing – review and editing (equal). **Emília Ondrušková:** formal analysis (equal), investigation (equal), writing – review and editing (equal). **Zuzana Jánošíková:** funding acquisition (lead), investigation (equal), project administration (equal), writing – review and editing (equal).

## Funding

This work was supported by the Slovak Research Grant Agency VEGA projects no. 2/0132/22 and 2/0034/25. The funding bodies had no role in the design of the study, data collection, analysis, interpretation of data, or writing of the manuscript.

## Ethics Statement

The authors have nothing to report.

## Conflicts of Interest

The authors declare no conflicts of interest.

## Supporting information


**Figure S1:** Relative abundance of fungal classes across individual *Pinus sylvestris* needle samples.

**Figure S2:** Relative abundance of fungal families across individual *Pinus sylvestris* needle samples.

**Figure S3:** Relative abundance of fungal genera across individual *Pinus sylvestris* needle samples.

**Table S1:** Sample‐level results of qPCR detection of *Dothistroma septosporum* in *Pinus sylvestris* needle samples.

## Data Availability

All data supporting the findings of this study are available within the manuscript. All sequence data were submitted to GenBank databases as part of BioProject PRJNA1455419. Any additional information is available from the corresponding author upon reasonable request.
